# Comparison of case and control groups in terms of postoperative complications, outcomes, and survival in total hip arthroplasty patients with and without COVID-19

**DOI:** 10.3389/fmed.2023.1231655

**Published:** 2023-11-23

**Authors:** Fadime Cinar, Semra Bulbuloglu

**Affiliations:** ^1^Division of Surgical Nursing, Nursing Department, Faculty of Health Sciences, Istanbul Nisantasi University, Istanbul, Türkiye; ^2^Division of Surgical Nursing, Nursing Department, Faculty of Health Sciences, Istanbul Aydin University, Istanbul, Türkiye

**Keywords:** COVID-19, geriatric patients, postoperative complications, survival, total hip arthroplasty

## Abstract

**Introduction:**

Total hip aarthroplasty (THA) is not performed worldwide as an emergency surgical procedure and has often been postponed during the COVID-19 pandemic. The hypothesis of this study was that COVID-19 caused the extra burden and mortality of THA patients. This study aimed to investigate the problems caused by comorbid COVID-19 in addition to the current clinical status in patients who had undergone THA.

**Materials and methods:**

A case–control group study was conducted with the participation of patients with COVID-19 and without COVID-19 who had undergone THA and were hospitalized in an orthopedic clinic and intensive care unit at a research and training hospital. IBM Statistical Package for Social Sciences 25.0 was used for data analysis.

**Results:**

A total of 291 patients who had undergone THA were included in the study: 161 patients with COVID-19 constituted the control group and 130 patients without COVID-19 constituted the case group. In this study, the authors found that THA patients with COVID-19 had higher rates of nausea, vomiting, diarrhea, musculo-articular pain, and headache compared to those without COVID-19, and the difference was statistically significant (*p* < 0.01); 17.7% of the patients with COVID-19 died, and delirium developed in 35.4%. The difference between the case and the control groups was statistically significant in terms of delirium and death (*p* < 0.01).

**Discussion:**

COVID-19 increases the risk of delirium and death in THA patients, as well as extending the hospital stay. The fact that the pandemic is not over yet and that despite vaccination, COVID-19 continues to have its devastating effects experienced, especially by individuals in their advanced age.

## Introduction

In cases where conservative treatment options do not prove beneficial, total hip arthroplasty (THA) is carried out in order to treat joint osteoarthritis, alleviate pain, and eliminate mobility restriction (1). It has been reported that 44 per 100,000 individuals in Turkey have undergone THA. It has also been reported that the frequency of applying THA is 292/100,000 in Switzerland, 283/100,000 in Germany, and 276/100,000 in Austria (2). Frequently observed complications in the post-THA period can be listed as infection development, thromboembolic event, heterotopic ossification, dislocation, reoperation, and nerve injury (3). Despite the frequently observed complications, it is known that THA stops the pain and increases the functionality of the hip. Moreover, it is not an emergency intervention (4). THA not being an emergency intervention is a controversial issue. Considering the bio-psychosocial result of the severe pain and restricted mobility to which the patient is exposed, it can be stated that THA must be included in the top emergency surgical interventions. Throughout the COVID-19 pandemic, healthcare services have been interrupted at a significant level (5). In the COVID-19 pandemic process, THA operations have been reduced considerably as it is recognized to be an elective surgical intervention. THA is a major bone surgery, the recovery time can be long, it serves the backward geriatric population, and the possibility of postoperative complications is high. Having COVID-19 concurrently with THA increases the burden of disease.

COVID-19that causes severe acute respiratory problems first emerged in Wuhan city of China in December 2019, and it is a fatal disease that has caused a pandemic at a global scale (6, 7). In addition to various treatment protocols applied in order to weaken the pathogenic mechanism of COVID-19, vaccination is also applied. In spite of vaccination campaigns all over the world, it still continues to lead to horrible consequences. In a study previously conducted, it was reported that 2,246 out of 1,228,664 individuals who had their first two dosages of vaccination were infected by COVID-19. In the same study, it was reported that 36 individuals who were infected by COVID-19 died and 189 experienced the disease at a severe level (8). The problems that are caused by COVID-19 are mostly fever, cough, shortness of breath, muscle pain, sore throat, nosebleed, chest pain, diarrhea, nausea and vomiting, dysphagia, central nervous system problems, and cerebrovascular events (9–13). Although vaccination is a unique product of today’s advanced technology and the best way to cope with COVID-19, it has been insufficient to prevent the death caused and complications from COVID-19.

Throughout the COVID-19 pandemic, there have been significant restrictions in applying THA, and THA has been interrupted in Turkey and all over the world. Therefore, we did not encounter any studies in which the problems experienced by patients who had undergone THA during the COVID-19 pandemic process were investigated. As a response to patients’ requirements and demands, certain health centers in Turkey were converted to COVID-19 pandemic hospitals where health service was provided to only COVID-19 patients. Large health institutions where surgical interventions were frequently performed in the pre-COVID-19 pandemic process continued their activities, and surgical interventions at these centers were not significantly interrupted. Thus, we had the opportunity to examine the effects of the COVID-19 pandemic on patients who had undergone THA. Exposure to COVID-19 may be associated with prolonged morbidity, higher mortality, and greater complications in THA patients. They have a very high risk of contracting COVID-19 during the perioperative period. In the study, we aimed to examine the problems caused by comorbid COVID-19 in patients who had undergone THA in addition to the current clinical conditions by comparing them with THA patients without COVID-19.

## Materials and methods

### Study design and participants

In the study, we compared the problems caused by COVID-19 in patients who had undergone THA in addition to the current clinical conditions of THA patients without COVID-19. The study with a case–control design was conducted in the orthopedics clinic and intensive care unit of a research and training hospital between October 2020 and May 2021.

### Ethical aspect of the study

Prior to the study, written permission and approval were taken from the relevant hospital’s head physician and Sabahattin Zaim University Ethics Committee (Decision No: E-20292139-050.01.04-424). In compliance with the Declaration of Helsinki, informed consent was taken from the participants.

### Study population and sample

The population of the study consisted of 291 patients who had undergone THA at a research and training hospital during the COVID-19 pandemic process after vaccination use became widespread. In order to calculate the sample size, G Power 3.1.9.7 software was used. Accordingly, the study sample was divided into two as the case group of 130 patients and the control group of 161 patients with 0.4 impact size, 0.04 margin of error, 0.95 confidence interval, and 95% power of representing the population. Fifty-four patients who did not meet the inclusion criteria and did not volunteer to participate in the study were excluded from the study sample.

### Determination of the case and control groups

In assigning the patients to the control and case groups, the purposive sampling method was used. Hip fractures were categorized according to the AO/OTA classification. The patients’ characteristics were presented in tables, and which of the two groups would be the case group or the control group was determined according to the inclusion and exclusion criteria. In the perioperative period, we assigned THA patients with positive COVID-19-polymerase chain reaction (PCR) test to the experimental group and those who were negative to the control group.

### Inclusion criteria

(i). Patients aged 18 years and above who volunteered to participate in the study were included in both groups.
(ii). Patients who had undergone THA were included in both groups.
(iii). THA patients who were in the perioperative process and in the acute phase of COVID-19 were assigned to the case group. THA patients who were not diagnosed with COVID-19 in the last 3 months and in the perioperative process were assigned to the control group.

### Exclusion criteria

(i). Patients below the age of 18 years who did not agree to participate in the study were excluded from both groups.
(ii). Patients who had not undergone THA were excluded from both groups, non-Turkish speakers and those having a communication barrier.
(iii). THA patients who were not in the acute phase of COVID-19 were excluded from the case group. Patients who were infected with and/or in the acute phase of COVID-19 and/or showed COVID-19 symptoms and/or tested COVID-19 positive in the last 3 months were excluded from the control group.

### Data collection tools

For data collection of the study, patients’ characteristics and COVID-19 Information Form was used. Information about the said form is presented below.

### Patients’ characteristics and COVID-19 information form

In the Patients’ Characteristics and COVID-19 Information Form (COVID-19-related symptoms, PCR test result), there are questions inquiring about the patients’ age, sex, type of fracture, chronic diseases, postoperative complications, and outcomes.

### Charlson comorbidity index

In the Charlson comorbidity index (CCI), a total of 19 comorbidities and conditions are evaluated and a score is created accordingly. It is an index based on which a 1-year mortality prediction is made. Various diseases and conditions are categorized in CCI. It receives scores between 1, 2, 3, and 6 depending on the risk level. Then, the total score predicts mortality with the summed scores. CCI was developed by Charlson (14).

### Statistical evaluation of the data

The study data were evaluated using Statistical Package for Social Sciences version 25.0 software for Windows (IBM SPSS Statistics for Windows, Version 25.0. Armonk, NY: IBM Corp., USA). The normality assumption for quantitative variables was tested with Kolmogorov–Smirnov test and normal distribution was present. Descriptive statistics of the variables were given as mean ± standard deviation (S.D.) and *n* (%). For the univariate analyses of the variables in the study, chi-square test and Student’s t test for independent groups were used, depending on the type of the variable and the availability of assumptions. The odds ratio of developing delirium between COVID-19-positive and COVID-19-negative THA patients was calculated with the odds ratio (OR). With Kaplan–Meier analysis, the probability of survival was calculated. Statistical significance was set at value of *p* ≤ 0.01 and value of *p* ≤ 0.05.

## Findings

In Table 1, THA patients’ descriptive characteristics and COVID-19-related symptoms are presented. A total of 291 patients who had undergone THA were included in the study. 161 patients without COVID-19 constituted the control group, while 130 patients with COVID-19 formed the case group. The patients in the control group did not have any infections in the perioperative process. However, 7.7% of the patients in the case group were infected in the preoperative process, and 92.3% were detected to have been infected in the postoperative period. There was no statistically significant difference between the experimental and control groups in terms of age, sex, and American Society of Anesthesiology (ASA) scoring, type of fracture (*p* > 0.05). Differently in the case group, all patients tested COVID-19 positive, and they had symptoms of fever, dyspnea, cough, chest pain, and muscle weakness. The rate of nausea and vomiting, diarrhea, musculo-articular pain, and headache in patients with COVID-19 was higher, and the difference was statistically significant (*p* < 0.01) (Table 1).

**Table 1:** Comparison of descriptive characteristics and COVID-19-related symptoms in THA patients (*N* = 261).

Characteristics	Category	Without COVID-19 *n* = 161	With COVID-19 *n* = 130	Homogeneity test and value of *p*
Sex *n* (%)	Female Male	30 (18.6) 131 (81.4)	59(45.3) 71 (54.7)	χ^2^ = 24.244 *p* = 0.001**
Age (year)	XX ± SD	66.30.80 ± 6.71	72.19 ± 6.31	0.009** t = 7.511
COVID-19 diagnosis *n* (%)	Preoperative Postoperative	–	10 (7.7) 120 (92.3)	–
ASA scoring	II III	20 (12.42) 141 (87.58)	17 (13.07) 113 (86.93)	χ^2^ = 0.991 *p* = 0.074
Type of fracture (*n*, %)	Femoral neck Intertrochanteric Subtrochanteric	60 (36.64) 66 (41.61) 35 (21.75)	40 (30.8) 68 (52.3) 22 (16.9)	χ^2^ = 1.178 *p* = 0.187

COVID-19-related symptoms	Category	Without COVID-19 *n* = 161	With COVID-19 *n* = 130	Test and value of *p*
Fever *n* (%)	No Yes	–	59 (45.3) 71 (54.7)	–
Dyspnea *n* (%)	No Yes	–	90 (69.2) 40 (30.8)	–
Cough *n* (%)	No Yes	–	80 (61.5) 50 (38.5)	–
Chest pain *n* (%)	No Yes	–	110 (84.6) 20 (15.4)	–
Muscle weakness *n* (%)	No Yes	–	65 (50.0) 65 (50.0)	–
Nausea *n* (%)	No Yes	116 (72.0) 45 (28.0)	56 (43.1) 74 (56.9)	χ^2^ = 7.262 *p* = 0.009**
Vomiting *n* (%)	No Yes	137 (85.0) 24 (15.0)	58 (44.7) 72 (55.3)	χ^2^ = 31.366 *p* = 0.000**
Diarrhea *n* (%)	No Yes	128 (79.5) 33 (20.5)	71 (54.6) 59 (45.4)	χ^2^ = 36.456 *p* = 0.000**
Musculo-articular pain *n* (%)	No Yes	106 (65.8) 55 (34.2)	59 (45.4) 71 (54.6)	χ^2^ = 3.802 *p* = 0.004**
Taste impairment *n* (%)	No Yes	–	53 (52.2) 77 (47.8)	**–**
Smell disorder *n* (%)	No Yes	–	35 (41.0) 95 (59.0)	–
Headache *n* (%)	No Yes	103 (63.9) 58 (36.1)	17 (13.1) 113 (86.9)	χ^2^ = 19.799 *p* = 0.000**

XX, Mean; SD, standard deviation; *p < 0.05, **p < 0.01; *χ*^2^, Pearson chi-square test; *t*, Student’s *t*-test.

ASA, American Society of Anesthesiology.

In Table 2, a comparison of the presence of chronic diseases, postoperative complications, and patient outcomes in the case and control groups is presented.

**Table 2:** Comparison of chronic diseases, postoperative complications and outcomes in THA patients with and without COVID-19 (*N* = 261).

Previous comorbid diseases^a^	Category	Without COVID-19 *n* = 161 *n*/%	With COVID-19 *n* = 130 *n* /%	Test and value of *p*
Chronic disease (mild kidney, liver, and thyroid diseases)	No Yes	88 (54.6) 73 (45.4)	8 (6.2) 122 (93.8)	χ^2^ = 53.921 *p* = 0.000**
Hypertension	No Yes	75 (46.6) 86 (53.4)	52 (40) 78 (60)	χ^2^ = 1.268 *p* = 0.286
Diabetes mellitus	No Yes	75 (46.4) 86 (53.6)	58 (44.6) 72 (55.4)	χ^2^ = 0.202 *p* = 0.813
Coronary artery disease/heart failure	No Yes	75(46.6) 86 (53.4)	48 (36.9) 82 (63.1)	χ^2^ = 11.847 *p* = 0.062
Cardiovascular disease	No Yes	111 (68.9) 50 (31.1)	56 (43.3) 74 (56.7)	χ^2^ = 4.488 *p* = 0.038*
Respiratory disease	No Yes	139 (86.3) 22 (13.7)	71 (54.6) 59 (45.4)	χ^2^ = 36.027 *p* = 0.000**

Postoperative complications^a^	Category	*n*/%	*n*/%	*n*/%
Pulmonary complication	No Yes	145 (90.1) 16 (9.9)	38 (29.2) 92 (70.8)	χ^2^ = 47.513 *p* = 0.000**
Cardiac complication	No Yes	145 (90.1) 16 (9.9)	30 (23.1) 100 (76.9)	χ^2^ = 9.330 *p* = 0.003**
Neurological complication	No Yes	142 (88.2) 19 (11.8)	11 (8.5) 119 (91.5)	χ^2^ = 21.175 *p* = 0.000**
Hemorrhagic complication	No Yes	161 (100) 0 (0)	121 (93.1) 9 (6.7)	χ^2^ = 11.502 *p* = 0.001**
Thrombotic complication	No Yes	145 (90.1) 16 (9.9)	30 (23.1) 100 (76.9)	χ^2^ = 2.719 *p* = 0.003**

Postoperative outcomes^a^	Category	*n*/%	*n*/%	*n*/%
Number of patients taken to intensive care (Intubated)	No Yes	148 (91.9) 13 (8.1)	62 (47.7) 68 (52.3)	χ^2^ = 70.057 *p* = 0.000**
Death	No Yes	157 (97.5) 4 (2.5)	107(82.3) 23 (17.7)	χ^2^ = 30.929 *p* = 0.000**
Delirium	No Yes	152 (94.4) 9 (5.6)	84(64.6) 46(35.4)	χ^2^ = 41.654 *p* = 0.000**
Time of death (until day 30)	(XX ± SD)	11.50 ∓ 2.8	8.08 ∓ 1.56	*t* = 6.796 *p* = 0.001**
Number of days of stay in the ward (including the day of admission and discharge)	(XX ± SD)	7.18 ± 5.22	9.46 ± 8.10	*t* = 9.668 *p* = 0.054**
Surgical operation time (day)	(XX ± SD)	205.98 ± 45.13	167.53 ± 56.03	*t* = 6.483 *p* = 0.000**

XX, Mean; SD, standard deviation; *p < 0.05, **p < 0.01; *χ*^2^, Pearson’s chi-square test; *t*, Student’s *t*-test.

a Diagnosed by a physician specialized in the field.

The perioperative chronic diseases, postoperative complications, and a 30-day follow-up of the patients included in the study were compared in both COVID-19-positive and COVID-19 negative groups. Chronic disease, cardiovascular disease, and respiratory disease (*p* < 0.05) were statistically different (*p* < 0.05) and more in those who were COVID-19 positive. Pulmonary, cardiac, hemorrhagic, thrombotic, and neurological complications were significantly different in both groups (*p* < 0.05). The difference resulted from those who were COVID-19 positive. The postoperative CCI score was higher in patients with COVID-19, and this difference was statistically significant (*p* < 0.05). In addition, the number of patients admitted to the intensive care unit, the number of days of hospitalization, and the number of patients who died in the postoperative 30-day follow-up, the average number of days of death were significantly different between those who were COVID-19 positive and those who were COVID-19 negative. The difference resulted from those who were COVID-19 positive. Following THA, 52.3% of the patients with COVID-19 were transferred to ICU, while this rate was 8.1% for those without COVID-19. Similarly, 17.7% of the patients with COVID-19 died, and delirium developed in 35.4%; 2.5% of those without COVID-19 died, and delirium developed in 5.6%. The difference between the case and the control groups was statistically significant in terms of delirium and death (*p* < 0.01) (Table 2).

According to the results of multivariate logistic regression analysis, age was 1.2 times (95% CI:1.13–1.27), presence of chronic disease (diabetes mellitus, hypertension, obesity, etc.) was 8 times (95% CI:4.41–14.55%), cardiovascular disease was 1.68 times (95% CI:1.03–2.71), respiratory disease was 5.25 times (95% CI:2.97–9.25), pulmonary complication was 2.23 times (95% CI:1.62–3.07), cardiac complication was 2.71 times (95% CI:1.40–5.25), thrombotic complication was 2.73 times (95% CI:1.48–5.32), 30-day death was 5.9 times (95% CI:1.88–18.48) higher in THA patients with COVID-19, and the mean day of death within 30 days was 7.8 times (95% CI:2.89–23.58) lower in those with COVID-19 (Table 3).

**Table 3:** Regression analyses for chronic diseases, postoperative complications, and post-operative outcomes in THA patients with COVID-19 (*N* = 261).

Predictors in the model	Odds ratio (95% CI)	Value of *p*
*Characteristics*		
Sex	2.01 (0.8–3.6)	*p* = 0.014*
Age	1.2 (1.13–1.27)	*p* = 0.000**
COVID-19 diagnosis	NA	NS
ASA scoring	NA	NS
Type of fracture	NA	NS
*Previous comorbid diseases*		
Chronic disease (Mild kidney, liver, and thyroid diseases)	8.01 (4.41–14.55)	*p* = 0.000**
Hypertension	NA	NS
Diabetes mellitus	NA	NS
Coronary artery disease/heart failure	NA	NS
Cardiovascular disease	1.68 (1.03–2.71)	*p* = 0.035*
Respiratory disease	5.25 (2.97–9.25)	*p* = 0.001**
*Postoperative complications*		
Pulmonary complication	2.23 (1.62–3.07)	*p* = 0.003**
Cardiac complication	2.71 (1.40–5.25)	*p* = 0.000**
*Neurological complication*		
Hemorrhagic complication	0.65 (0.21–0.957)	*p* = 0.995**
Thrombotic complication	2.73 (1.48–5.32)	*p* = 0.003**
*Follow-up*		
Number of patients taken to intensive care	12.48 (6.43–24.23)	*p* = 0.003**
Death	5.90 (1.88–18.48)	*p* = 0.002**
Delirium	9.24 (4.32–19.82)	*p* = 0.000**
Time of death (until day 30)	7.84 (2.89–23.58)	*p* = 0.031**
Number of days of stay in the ward (including the day of admission and discharge)	0.2 (0.1–0.8)	*p* = 0.008**
Surgical operation time (day)	3.12 (1.3–6.1)	*p* = 0.011*

*p < 0.05, **p < 0.01; NA, not applicable; NS, not significant (*p* < 0.05).

**Figure 1:**
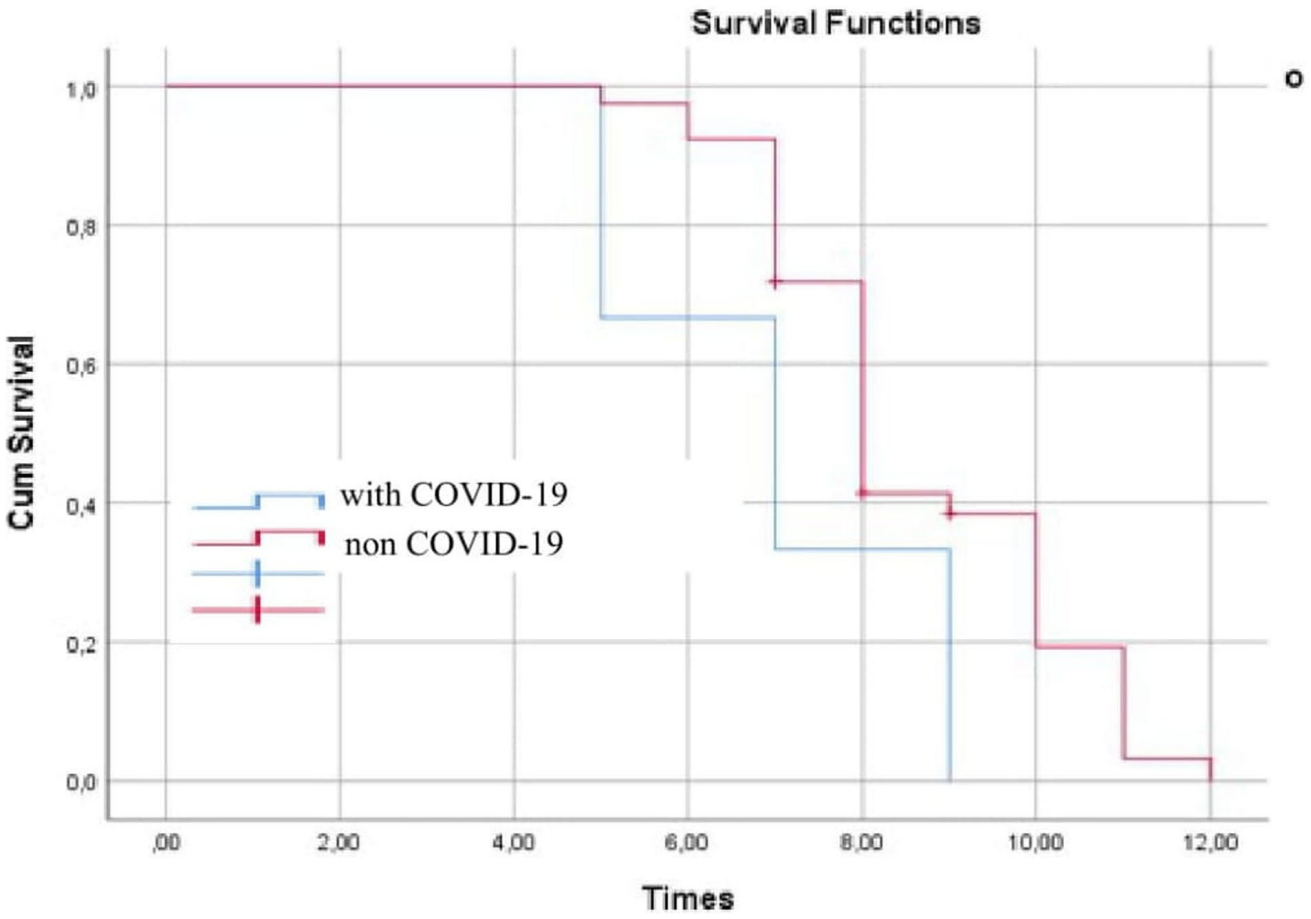
30-day mortality curve of patients with COVID-19 who had undergone THA.

A survival probability assessment in delirious and non-delirious COVID-19 patients along with time of death (until day 30) was performed using the Kaplan–Meier curve (Figure 1). It presents a statistically significant difference in 30-day survival between both groups in favor of patients without COVID-19 (*p* < 0.01). THA patients with COVID-19 had to face death earlier.

**Figure 2:**
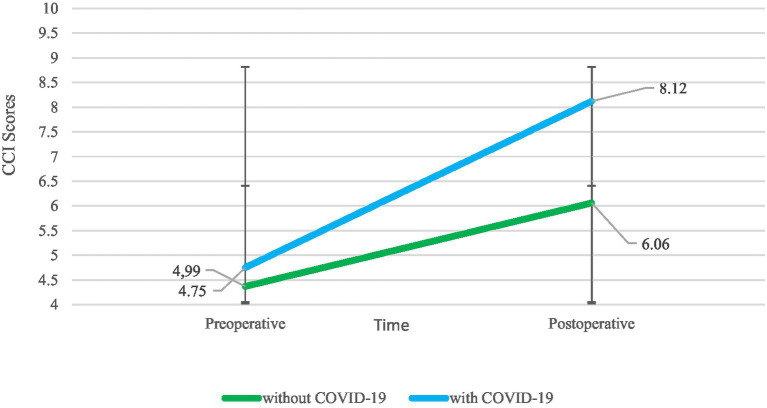
Change in pre/postoperative CCI scores of COVID-19-positive and COVID-19-negative patients with THA.

Figure 2 shows the CCI scores of COVID-19-positive and COVID-19-negative patients before and after THA. The CCI score of THA patients with COVID-19 showed a dramatic upward trend after THA.

## Discussion

In the COVID-19 pandemic process, THA cases were significantly interrupted in Turkey and worldwide. Toward the second half of 2020, THA cases resumed again. In the COVID-19 pandemic, THA surgeries cause very serious consequences. In a study conducted, in THA patients with comorbid COVID-19, pneumonia, anemia that required one unit of blood transfusion, and acute renal injury were detected in the postoperative process. In the same study, the duration of hospital stay was reported to be 7 days (15). During the peak of the pandemic, spinal surgery was performed on 246 patients, including 6 patients with positive COVID-19 tests. In the same study, the importance of maintaining spinal surgery in times of health crisis was emphasized, thanks to minimally invasive methods, multidisciplinary team members including anesthesiologists, surgeons and infection specialists, and the technical possibilities of treatment (16). In the present study, pulmonary, thrombotic, hematologic, neurological, and cardiovascular problems developed in THA patients with COVID-19. The hospital stay duration of THA patients without COVID-19 was determined to be 7.18 ± 5.22, while this duration was found as 9.46 ± 8.10 in THA patients with COVID-19. The results of the present study are similar to the results found in the literature.

THA is a major surgical procedure that combines anesthesia, drug therapy, tissue trauma, blood loss, and body temperature changes. In THA patients, a series of events develop during the perioperative process, metabolic changes occur, and stress response begins simultaneously. Exposure to surgical trauma induces a neurohumoral response and increases catabolism. COVID-19 increases the stress level and catabolism in THA patients and makes it difficult to cope with the symptoms and complications of surgery. The mean age of patients who underwent THA has been reported as 70 years in the literature (17). In the present study, the mean age of THA patients without COVID-19 was determined as 66.30.80 ± 6.71, while the mean age of THA patients with COVID-19 was found as 72.19 ± 6.31. As age increases, the vulnerability of patients to COVID-19 and its harmful effects can increase as well. In this regard, the present study complies with the literature.

Complications caused by general anesthesia (nausea and vomiting, diarrhea, musculo-articular pain, and headache) in patients with COVID-19 were higher, and the difference was statistically significant. Patients who tested negative for COVID-19 had less similar complications. From our findings, we can conclude that COVID-19 increases the incidence or severity of complications caused by general anesthesia. In a study conducted in the literature with the participation of 388,424 THA patients, postoperative delirium incidence was determined as 0.9%. In the same study, postoperative delirium was associated with problems determined in the preoperative period, such as advanced age, neurological disorders, fluid and electrolyte disorders, diabetes, weight loss, anemia, coagulopathy, hypertension, congestive heart failure, and pulmonary circulation disorders (18).

In a previous study, it was determined that the average hospital stay and time to surgery in 3 patients with COVID-19 who required emergency surgical care were not different from other patients (19). In the present study as well, similar comorbidities were determined in patients with and without comorbid COVID-19 in the preoperative period. However, the rate of development of large-scale problems in the postoperative process and those who died was higher in THA patients with COVID-19. In the present study, in the postoperative process, 17.7% of THA patients with COVID-19 died, and delirium developed in 35.4%. 2.5% of those without COVID-19 died, while delirium developed in 5.6%. The difference between the case group and the control group was statistically significant in terms of delirium and death (*p* < 0.01). Based on the results obtained in the present study, it is possible to claim that THA patients with COVID-19 had higher risks in terms of delirium and that COVID-19 increased the risk of death in THA patients.

The mean age of the patients who had undergone THA was above 65 years. Advanced age is an important predictor of the increased severity of COVID-19 symptoms. In addition, comorbidity was present in the great majority of aged patients. It has been reported in the literature that the clinical course of cardiovascular disease (CVD), diabetes mellitus (DM), neurological disorder, and COVID-19 in individuals with advanced age is worse (20–22). In the present study, the complication development rate of the said diseases was higher in COVID-19 patients in the postoperative THA period, and the difference was statistically significant (*p* < 0.05). The development risk of central nervous system-related disorders is higher in individuals with COVID-19 (23). It has been reported in the literature that after the acute phase of COVID-19 is overcome, neurological disorders emerge in the ensuing periods (24).

Common major neurological problems caused by COVID-19 include acute cerebrovascular events, increase in the risk of stroke and peripheral nerve diseases, Guillain–Barrè syndrome, as well as intracerebral hemorrhage, central nervous system (CNS) problems, confusion, and convulsive seizure (25, 26). The degree and severity of the COVID-19 disease process is significantly associated with the development of psychiatric and neurological disorders. Previous studies have reported increased serum levels of pro-inflammatory cytokines, interleukin (IL)-6 and IL-8, in elderly hip fracture patients with delirium (27, 28). In addition, increased serum interferon gamma (IFN-γ) and decreases in insulin-like growth factor (IGF)-1 and IL-1 have been associated with delirium in elderly patients receiving medical treatment (29, 30). Neurological vulnerability of aged individuals who have weak immune systems and comorbid or chronic diseases increases (31–33). This group of patients may not tolerate the burden of THA. In fact, in the present study, the rate of death and delirium in THA patients with COVID-19 was strikingly high. While there was a significant decrease in the incidence of patients requiring orthopedic and trauma surgery during the peak months of the COVID-19 pandemic, only an increase was observed in the number of elderly femoral fractures (34). It was very difficult not to intervene in the elderly THA patients during the pandemic process. There would be a significant increase in the number of patients toward the end of the pandemic, and most of the THA patients had the potential to require urgent surgical intervention.

Variants of concern (VOCs) began to emerge as a result of the antigenic drift of Sars-CoV-2. Variants P.1 (in Brazil) (35), B.1.351 (in South Africa) (36), B.1.1.7 (in the United Kingdom) (37), and B.1.617/B.1.617.2 (in India) (38) are more transmissible and increased the severity of the COVID-19 pandemic when they emerged. That’s why it’s so important to slow the transmission of COVID-19 and take safety precautions to prevent infection (39). It is currently unknown whether the antibody responses of THA patients who have recovered from COVID-19 can protect against reinfection with emerging VOCs. Vaccines developed against COVID-19 are based on the D614G strain. If VOCs have conformational changes other than the spike protein, reduced neutralizing antibody production will pose a significant problem (40, 41). Moreover, P.1, B.1.351, B.1.1.7, and B.1.617/B.1.617.2 variants are resistant to some antibodies used for SARS-CoV-2 treatment (42). In Turkey, it is noteworthy that the B.1.617.2 variant was dominant between April and June 2021. It is known that before April 2021, the B.1.1.7 variant was mostly dominant (43–45). COVID-19 continues to pose a significant danger for THA patients due to problems, such as the risk of death, difficulties in its management, and unknown course of events. The results of our study provide important data that COVID-19 worsens patient prognosis in THA patients. In this respect, it is very valuable and will lay the groundwork for the management of possible future effects of COVID-19 VOCs. The study had certain limitations; the first limitation is that a brain scan was not performed on the patients through neuro-imaging methods. The other limitations of the study are that the patients’ interleukin and other cytokine values were not examined and that COVID-19 is a respiratory-related disease.

## Conclusion

The study has included the problems caused by comorbid of COVID-19 in addition to the current clinical status in patients who had undergone THA. There is an ongoing controversy about THA being considered in the category of elective surgery instead of the category of emergency surgery and being postponed in the COVID-19 pandemic period, although it is known that it plays a significant role in increasing the independence of aged individuals and their quality of life. In our study, postoperative pulmonary, cardiac, hemorrhagic, and thrombotic complications were much higher in COVID-19-positive THA patients. In addition, the number of patients admitted to the intensive care unit, the number of days of hospitalization, and the number of patients who died in the postoperative 30-day follow-up, the average number of days to death were common in THA patients with COVID-19. The present study is an important source of information in this regard, and results that could justify the postponement of THA in the COVID-19 pandemic process were obtained. Increased risk of death and delirium in THA patients with COVID-19 and extended morbidity and hospital stay lead to late recovery. The fact that THA patients, whose quality of life and independence we aim to increase, were in need of hospital care for a long time in the postoperative process due to the effect of the COVID-19 pandemic suggests that THA should be avoided as much as possible.

## Data availability statement

The original contributions presented in the study are included in the article/supplementary material, further inquiries can be directed to the corresponding author.

## Ethics statement

The studies involving human participants were reviewed and approved by the Sabahattin Zaim University Ethics Committee (Decision No: E-20292139-050.01.04-424). The patients/participants provided their written informed consent to participate in this study.

## Author contributions

SB: Writing – original draft, Writing – review & editing, Methodology, Supervision, Project administration, Investigation, Funding acquisition, Resources, Visualization, Software. FC: Writing-original draft, Data curation, Conceptualization, Formal analysis, Validation, Funding acquisition, Resources, Visualization, Software.
